# Embodied Emotion Recognition Based on Life-Logging

**DOI:** 10.3390/s19235308

**Published:** 2019-12-02

**Authors:** Ayoung Cho, Hyunwoo Lee, Youngho Jo, Mincheol Whang

**Affiliations:** 1 Department of Emotion Engineering, University of Sangmyung, Seoul 03016, Korea; joa6391@gmail.com (A.C.); lhw4846@naver.com (H.L.); 2 Team of Technology Development, Emotion Science Center, Seoul 03044, Korea; imzeus05@gmail.com; 3 Department of Intelligence Informatics Engineering, University of Sangmyung, Seoul 03016, Korea

**Keywords:** embodied emotion, causality, life-logging, photoplethysmogram (PPG), global positioning system (GPS), ambient noise

## Abstract

Embodied emotion is associated with interaction among a person’s physiological responses, behavioral patterns, and environmental factors. However, most methods for determining embodied emotion has been considered on only fragmentary independent variables and not their inter-connectivity. This study suggests a method for determining the embodied emotion considering interactions among three factors: the physiological response, behavioral patterns, and an environmental factor based on life-logging. The physiological response was analyzed as heart rate variability (HRV) variables. The behavioral pattern was calculated from features of Global Positioning System (GPS) locations that indicate spatiotemporal property. The environmental factor was analyzed as the ambient noise, which is an external stimulus. These data were mapped with the emotion of that time. The emotion was evaluated on a seven-point scale for arousal level and valence level according to Russell’s model of emotion. These data were collected from 79 participants in daily life for two weeks. Their relationships among data were analyzed by the multiple regression analysis, after pre-processing the respective data. As a result, significant differences between the arousal level and valence level of emotion were observed based on their relations. The contributions of this study can be summarized as follows: (1) The emotion was recognized in real-life for a more practical application; (2) distinguishing the interactions that determine the levels of arousal and positive emotion by analyzing relationships of individuals’ life-log data. Through this, it was verified that emotion can be changed according to the interaction among the three factors, which was overlooked in previous emotion recognition.

## 1. Introduction

The theory of the embodied mind has recently emphasized that emotion should be conceptualized as being operated by the inter-connectivity of the body, the behavior, and the environment because the intrinsic function of the emotion is for its adaptive survival in the environment [1,2]. Human physiological changes and behaviors have been dependent with the environment, and the interactions between them is a mechanism to cope with the environment [3]. Most scholars have agreed that there are correlations among the physiological response, behavior, and environment. Nevertheless, emotion has been recognized by fragmentary independent variables without consideration of the relationships among the three main factors. These are, the physiological response, behavior, and environmental factors. Therefore, it has been primitive that a heuristic understanding of the embodied emotion is missing connections among the three main factors. Moreover, the embodied emotions recognized in the laboratory have been difficult to apply to real life, due to its limitation, compared to the experiences in a complex, real-life environment. Ecological validity has been undermined by recognizing emotion in laboratory settings. Emotional expressions tend to be reduced due to social desirability [4]. Therefore, a field study is necessary to test the feasibility that emotional factors measured in laboratory settings are applicable in real environment [5,6,7].

Field studies of emotion recognition have tried to collect data and measure emotion using wearable sensors or smartphone [4,8,9]. Life-logging applications particularly hold for investigations conducted in the field. An increased use of wearable devices and self-tracking behaviors has been highlighted [10]. Life-logging is the process of automatically recording an aspects of one’s life in digital form [11]. They have mainly measured autonomic nervous system (ANS) for analyzing the physiological response in daily life [12,13,14,15,16,17]. The ANS included activities of sympathetic and parasympathetic nervous system, which has been measured by the responses of cardiovascular, respiratory, and electrodermal [18,19]. In particular, the photoplethysmoogram (PPG) has been increasingly measured from portable devices according to the commercialization of wearable devices, such as smart watches [14,16]. Therefore, the PPG signal is attracting attention as a measure of ANS that can be measured in daily life.

Behavioral patterns associated with emotions have based on individual’s own mobility patterns in life-logging studies. Stress and depression have been correlated with smaller variation of mobility [20,21]. The individual’s own movement patterns can be measured by the global positioning system (GPS) [21,22]. The GPS can be easily measured with sensors built into the smartphone. Emotions have been inherent in physiological mechanisms to adapt to environment [23]. Therefore, emotions have been affected by environmental factors.

The environment changes the physiological responses and human behavior according to emotions, and vice versa [24]. It also affects emotions. Embodied emotions are highly related to the environmental factors, such as sound [25,26] and exposure to ambient noise in daily life has been reported to affect negative emotions and arousal [27,28,29]. Louder or long-lasting noise has been reported to negatively impact emotion. Also, uncontrollable noise has been reported to have more emotional impact. On the other hand, there were the results that the white noise had a positive effect [30,31]. Schuller et al. [32] suggested that arousal is highly correlated with loudness and valence is negative correlated with spectral flux and spectral harmonicity. The arousal and valence levels are the dimension of the Russell’s emotional model [33]. These results indicate that ambient noise gives physical and emotional impact on humans and it could be one of the factors to induce emotions. Moreover, field studies of real-time noise monitoring suggested that environmental noise can be measured using smartphones [34,35,36]. Therefore, it can be easily measured in life-logging.

Despite the proposed application of emotion recognition in real-life, there is still little research, which is often overlooked for emotion recognition in real-time, by considering three factors. Therefore, this study attempts to recognize the emotions of the interactions of the three factors in real-life: Physiological responses by measuring PPG, behavior by measuring GPS, and the ambient noise as an environmental factor by measuring sound. The contributions of this study can be summarized as follows: (1) The emotion was recognized in real-life for more practical applications; (2) the proposed method analyzed the interactions of more causes of emotional determination compared with the previous emotion recognition method that employs fewer factors.

## 2. Method

### 2.1. Hypothesis

This study hypothesized that the interactions among the physiological response, the behavioral pattern, and the ambient noise would differ in the emotional arousal and valence.

### 2.2. Participant

Seventy-nine participants (35 males) without cardiovascular disease were selected by convenience sampling. Their average age was 23(±3). Everyone was given a detailed explanation and provided consent before the field test. Participants were compensated ($140.38) for their role in the field test.

### 2.3. Data Collection

**Figure 1:**
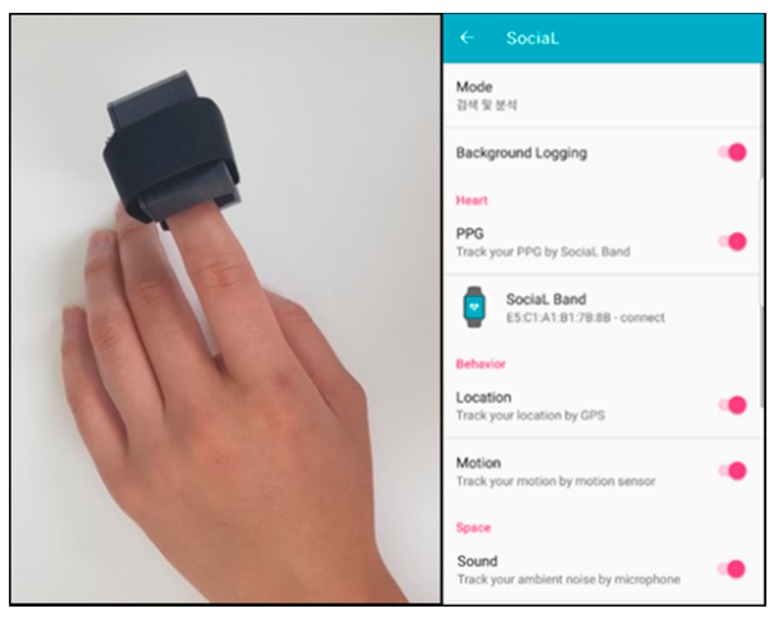
The wearable device for sensing photoplethysmogram (PPG) signals and the mobile application for PPG, photoplethysmogram (GPS), ambient noise, and self-report data acquisition.

Data were collected from a field test and not in a usual laboratory environment to ensure the authenticity of the physical experience and environmental factors of daily life. Seventy-nine participants were a part of this field test, which lasted 5 h, every day for 2 weeks, including the weekends. They were given a wearable device and received guidance for the smartphone application developed for this field test. The participants were asked to wear their devices by connecting it to their smartphones throughout the field test. They wore the device throughout the fixed time from 12 pm to 6 pm daily. These 5 h may be a working time or a rest time depending on the participant, but only the people who agreed to continue to measure data during this time participated in the experiment. A notification function in the application was developed to ensure that measurements are taken continuously throughout the experiment without missing data. The application sent a notification to the researcher when there was a lost connection with the sensor or data was not measured for a certain period. If the researcher received the notification from the application, the researcher asked the participant to continuously measure the data via messenger. The collected data consisted of physiological and behavioral responses based on photoplethysmogram, global positioning system location, along with environmental factors based on ambient noise. Physiological responses were separately measured with the wearable device, and the behavioral along with environmental factors were measured with the GPS sensor and microphone embedded in the smartphone, respectively. To avoid disturbing daily activities as much as possible, the wireless PPG sensor, which can be measured with one finger, was worn on the infrequently used hand (mostly left hand) as shown in Figure 1. Also, the data from GPS and the surrounding environment were automatically collected by using the smartphone that the participants always possesses during daily activities. It provided convenience for the participants. The participants only had to connect the application and the sensor at the start of the experiment to collect data. 

Participants received an emotional assessment request from the application on time every hour. They answered two emotional questions about how arousal and how pleasant by assessing their overall emotional state during the previous hour, based on the point of emotional evaluation. These two questions are based on two independent dimensions, the arousal axis, and the valence axis, which constitute emotion in Russell’s two-dimensional circumplex model [33]. Russell’s model is one of the most representative emotional models and has been evaluated on a seven-point Likert scale when rating arousal and valence levels in other emotional assessment studies [37,38,39,40,41,42]. Therefore, in this study, participants self-reported their emotions on a seven-point scale. Specifically, the participant checked one of the radio buttons from 1 to 7 points in the application and clicked the submit button. As with other data, if a subject did not respond to an emotional assessment, the application detected it and alerted the investigator. When the investigator received such an alert, the investigator asked the subject to evaluate the emotion. This experimental procedure was approved by the Institutional Review Board of the Sangmyung University, Seoul, Korea (BE2017-22).

### 2.4. Measurement of Physiological Response by Analyzing HRV

#### 2.4.1. Recording and Signal Processing

The PPG signals were recorded between 50 and 90 Hz sampling rate with the wireless PPG sensing system (Emotion Science Research Center Inc., Seoul, Korea). Zero-padding and cubic spline interpolation were applicated to stabilize the sampling rate of data to 80 Hz. After the interpolation, Only the frequency components between 0.75 and 2.5 Hz corresponding to the ranges between 50 bpm and 150 bpm, respectively, were extracted by the Butterworth bandpass filter for noise cancelation. The peak was detected in the PPG raw signal by the peak detection algorithm. The peak to peak interval (PPI), which is the interval between detected peaks, was calculated by detecting the dominant frequency by Fast Fourier Transformation (FFT), while sliding the raw data signal accumulated for 120 s at intervals of 1 s.

#### 2.4.2. HRV Analysis in Time Domain

Beat per minute (BPM) was calculated by dividing the window size of 60 s with the peak to peak interval (PPI) as:

(1) $$BPM=60/\frac{1}{N}\overset{N}{\underset{i=1}{\sum }}PPI_{i}$$

where N was the number of raw PPG signal samples. All variables of the heart rate variability (HRV) were calculated by HRV analysis with the window size of 180 s and interval size of 60 s. The mean of the standard deviation (SDNN) of all PPI for all 3-min segments of the entire recording was calculated as:

(2) $$SDNN=\;SD\left(PPI\right)=\sqrt{\frac{1}{N-1}\overset{N}{\underset{I=1}{\sum }}{\left[Mean\left(PPI\right)-PPI_{i}\right]}^{2}}$$

where SD(PPI) is a standard deviation of PPI. The root mean square of differences between adjacent PPI (RMSSD) was calculated as:

(3) $$RMSSD=\sqrt{\frac{1}{N-2}\sum_{i=2}^{N}{\left(PPI_{i}-PPI_{i-1}\right)}^{2}}.$$

The proportion derived by dividing the number of interval differences of PPI greater than 50 ms by the total number of PPI (pNN50) as:

(4) $$pNN50=\frac{NN50\;count}{total\;NN\;count}$$

where NN50 count is number of PPI greater than 50ms and total NN count is the total number of PPI.

#### 2.4.3. HRV Analysis in Frequency Domain

Very low frequency (VLF) which is the power in the frequency range of 0.0033–0.4 Hz was analyzed as an indicator of sympathetic activity as:

(5) $$VLF=\sum_{i=\frac{0.0033}{df}}^{\frac{0.04}{df}}Power_{i},\;\;df=\frac{SamplingRate\left(PPI\right)}{Length\left(PPI\right)}=\frac{1}{Time}$$

where Power is the power spectrum analyzed PPI by FFT and df is frequency resolution. Low frequency (LF) which is the power in the frequency range of 0.04–0.15 Hz was analyzed as an indicator of both the sympathetic and the parasympathetic activity as:

(6) $$LF=\sum_{i=\frac{0.04}{df}}^{\frac{0.15}{df}}Power_{i}$$

High frequency (HF) which is the power in the frequency range of 0.15–0.4 Hz was analyzed as an indicator of parasympathetic activity as:

(7) $$HF=\sum_{i=\frac{0.15}{df}}^{\frac{0.4}{df}}Power_{i}.$$

The VLF, LF, and HF components were also analyzed as percentage and normalized values, respectively. The percentage of each variable were calculated by dividing each variable by the total power. Total power is a band of power spectrum range between 0.0033 and 0.4 Hz as:

(8) $$TotalPower=\sum_{i=\frac{0.0033}{df}}^{\frac{0.4}{df}}Power_{i}.$$

VLF(%) is VLF divided by the total power as:

(9) $$VLF\left(\%\right)=\frac{VLF}{TotalPower}.$$

LF(%) is LF divided by the total power as:

(10) $$LF\left(\%\right)=\frac{LF}{TotalPower}.$$

HF(%) is HF divided by the total power as:

(11) $$HF\left(\%\right)=\frac{HF}{TotalPower}.$$

The normalized variables were calculated by natural logarithm of VLF, LF, and HF. lnVLF is natural logarithm of VLF as:

(12) lnVLF=ln(VLF)

where ln is natural logarithm. lnLF is natural logarithm of LF as:

(13) lnLF=ln(LF)

lnHF is natural logarithm of HF as:

(14) lnHF=ln(HF).

VLF, LF, and HF were also calculated as ratios such as LF/HF ratio, VLF/HF ratio. The LF/HF ratio and VLF/HF ratio represent homeostasis of the sympathetic and parasympathetic activity [43] as:

(15) $$LF/HF\;ratio=\frac{LF}{HF}$$

(16) $$VLF/HF\;ratio=\frac{VLF}{HF}.$$

Peak power is the band of power spectrum range between −0.015 and 0.015 Hz based on peak Hz. The peak power is an indicator of homeostasis [43] as:

(17) $$PeakPower=\sum_{i=\frac{PeakHz-0.015}{df}}^{\frac{PeakHz+0.015}{df}}Power_{i}.$$

Peak Hz is a hertz of highest peak in power spectrum range of 0.04–0.26 Hz as:

(18) $$PeakHz=\;argmax\left(Power_{i}\right)\times df,\;\;\frac{0.04}{df}\leq i\leq \frac{0.26}{df}.$$

Coherence ratio is the peak power divided by difference of total power and peak power which is indicator of the emotional stability [43] as:

(19) $$Coherence\;Ratio=\frac{PeakPower}{TotalPower-PeakPower}$$

Dominant power is a power of highest peak in total power spectrum range of 0–0.5 Hz as:

(20) $$Dominant\;Power=Power_{argmax\left(Power\right)}$$

Dominant Hz is a hertz of highest peak in total power spectrum range of 0–0.5 Hz as:

(21) Dominant Hz=argmax(Power)×df

### 2.5. Measurement of Behavior Patterns by Analyzing GPS Location

GPS locations were measured in two states: Stationary and transition state. The GPS locations were classified into a stationary state or transition state were defined based on a distance by K-Means algorithm. The GPS location in the stationary state were calculated when the latitude and longitude have changed by less than 1 km per hour, and the GPS locations in the transition state were considered only more than 1 km per hour [21]. The six variables of behavioral patterns were defined by analyzing the GPS locations accumulated for 10 min at intervals of 1 min. Location Variance is the variability in a participant’s GPS location which is calculated by logarithm of sum of squares of latitude and longitude as,

(22) $$Location\;Variance=\log\left(\sigma_{lat}{}^{2}+\sigma_{lng}{}^{2}\right)$$

where $\sigma_{lat}{}^{2}$ is a sum of squares of latitude and $\sigma_{lat}{}^{2}$ is a sum of longitude. Number of Clusters is the number of location clusters found by the k-means algorithm. Entropy is a variability of the time spent at the location clusters as,

(23) $$Entropy=-\overset{N}{\underset{i=1}{\sum }}p_{i}\log\;p_{i}$$

where *i* was the location cluster, *N* was the number of clusters, and $p_{i}$ was the ratio of the time spent in the clusters. Circadian Movement is the regularity of moving pattern in daily life as,

(24) $$Circadian\;Movement=\log\left(E_{lat}+E_{lng}\right),\;E=\overset{N}{\underset{i=1}{\sum }}\frac{psd\left(f_{i}\right)}{\left(i_{i}-i_{N}\right)}$$

where f was a bin in frequency domain analyzed from GPS locations by least-squares spectral analysis, *N* was the number of frequency bins corresponding to 24-h periods, *i* was the index of frequency bin, and $psd(f_{i})$ was the power spectral density at frequency bin $f_{i}$. The logarithm was applied to correct the skewed distribution. Transition Time is the percentage of time during which a participant was in the non-stationary state. Total Distance is accumulation of distances between the location samples in kilometers taken by a participant as,

(25) $$\begin{array}{cc} Total\;Distance & =\overset{N-1}{\underset{i=1}{\sum }}111.19\times \frac{180}{\pi }\times \mathrm{acos}[\sin\left(lat_{i}\right)\times sin\left(lat_{i+1}\right)\\ & +\cos\left(lat_{i}\right)\times \cos\left(lat_{i+1}\right)\times \cos(lng_{i+1}-lng_{i})] \end{array}$$

where *i* was the GPS location, and *N* was the total number of GPS locations, and 111.19 was the constant for unit conversion from miles to kilometers.

### 2.6. Measurement of Environmental Factors by Analyzing Ambient Noise

The environmental factors based on ambient noise were analyzed by raw sound signals. The raw signals of ambient noise were recorded every second while accumulated for 5 s. The analyzed features of raw signals were classified with volume and frequency components which are sound amplitude and sound frequency. The sound amplitude was analyzed by averaging the measured the raw signal for 1 min as,

(26) $$SoundAmplitude=\frac{1}{m}\overset{m}{\underset{i=1}{\sum }}Amplitude_{i}$$

where *Amplitude* is amplitude of ambient noise, and *m* is a window size. The sound frequency was analyzed by dominant power spectrum in frequency domain.

(27) $$SoundFrequency=argmax\left(Power\right)\times df,\;df=\frac{SamplingRate\left(Amplitude\right)}{Length\left(Amplitude\right)}=\frac{1}{Time}.$$

### 2.7. Statistical Analysis

The relationships among the physiological response, the behavioral pattern, and the ambient noise, based on emotion, were analyzed in the following three steps. First, the pre-processing step that interpolates and normalizes data samples. In the second step, the correlations, between the 31 variables measured the physiological response, the behavioral pattern, and the ambient noise, which are analyzed by the multiple regression. Finally, the hypothesis of this study, which is that significant relationships, resulting from multiple regression, differ depending on the emotions, is verified by ANOVA. Since the above variables have different criteria, data interpolation and standardization were performed to compare among the variables. The data were interpolated by averaging the data and standardized by z-score. After data preprocessing, a multiple regression model was constructed by setting one of the above 31 variables as the dependent variable and the remaining 30 variables as independent variables. The same procedure was repeated for all variables that were not set as dependent variables.

Correlations between the dependent variable and independent variables were analyzed by multiple regression. Multiple regression analysis is convenient to analyze multiple independent variables for a dependent variable. In order to derive a significant correlation between the dependent variable and independent variables by multiple regression, the following constraints should be checked. The multi-collinearity means that there is a strong correlation among independent variables. The independent variables which have multi-collinearity should have been removed to prevent error. In order to this constraint, the variance inflation factor (VIF) which is an indicator of multicollinearity was checked that is less than 10. The second, autocorrelation was tested by Durbin-Watson statistics. The autocorrelation indicates a strong correlation among dependent variables. It was verified that there was not the autocorrelation since the Durbin-Watson statistics were more than 1 and less than 3. Third, the normality and homogeneity of the residuals were tested by the Kolmogorov-Smirnov test (*p* > 0.1) and Breusch-Pagan (*p* > 0.05). All assumptions for multiple regression were satisfied, therefore, this study analyzed the multiple regression models. Finally, the existence of any significant independent variable, which affect a dependent variable, was verified (*p* < 0.05). The suitability of the regression model was verified by the adjusted r-squared, which is more than 0.6. The standardized coefficients (β) obtained as a result of multiple regression are indicators of the influence of each independent variable on the dependent variable.

**Figure 2:**
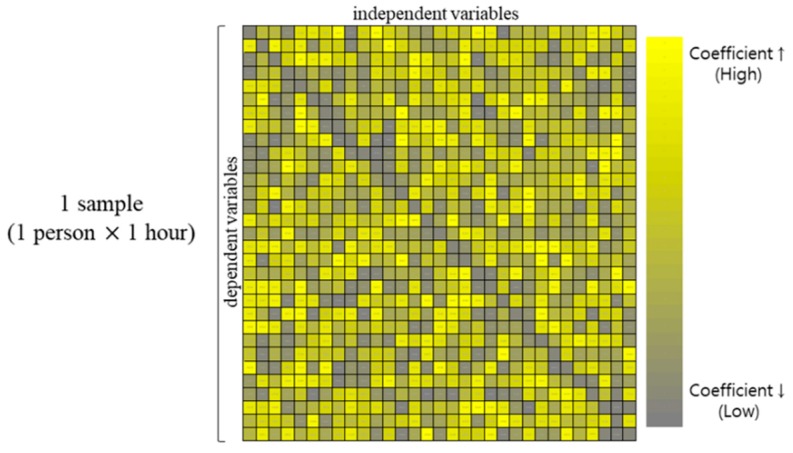
A sample of standardized coefficient matrix. The standardized coefficient which is beta (β) indicates the influence of each independent variable on the dependent variable.

**Figure 3:**
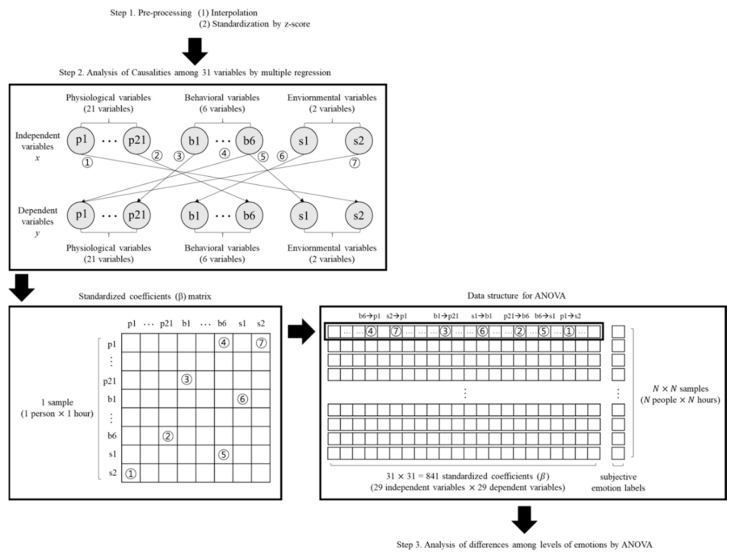
Data structures of each analysis step. Standardized coefficients as results of multiple regression were formed as a matrix. Map the subjective emotion labels to the standardized coefficients matrix to form a data structure for ANOVA.

The standardized coefficients for each person were stored in matrix form as shown in Figure 2. Then, the subjective emotion labels were mapped to the standardized coefficients matrix to form a data structure for ANOVA as shown in Figure 3. The emotion labels indicate the subjective questionnaire score, which is evaluated as seven points for two emotion questionnaires of arousal level and valence level. Samples that were analyzed 79 participants of standardized coefficient data mapped with emotional labels were analyzed for differences between arousal levels or valence levels respectively by ANOVA.

## 3. Results

Significant correlations among the physiological responses, the behavioral patterns, and the ambient noise were analyzed by multiple regression for two weeks of data samples of 79 participants’. An example of multiple regression results is shown in Table 1. The significant correlations derived from the multiple regression were analyzed by ANOVA. This analysis analyzed the difference between correlations depending on emotion levels. Specifically, we confirmed whether the standardized coefficients obtained through the multiple regression analysis were different according to the three emotion levels. The emotion was analyzed at the relevant time when a significant correlation was analyzed on the basis of the subjective emotion recorded from the participants. The emotion was recorded on seven-point scales according to the level of arousal, and valence, respectively. The level of arousal was classified into three levels based on the median of the assessment. The data larger than the median (5–7 points) were classified as arousal, the median (4-point) was neutral, and the data smaller than the average (1–3 points) were classified as relaxation. Likewise, 5–7 points of valence scores were classified into positive emotion, the median value (4-point) was into neutral, and 1–3 points were into negative emotion. The number of samples by arousal level was 1907 arousal samples, 1699 neutral, and 777 relaxation, respectively. While, 2300 samples were rated positive emotion, 1098 neutral, and 985 negative emotion. Specific descriptive statistics on mean and standard deviation of the standardized coefficients were presented according to the levels of arousal in Table 2. Descriptive statistics of the standardized coefficients according to the levels of valence were described in Table 3.

**Figure 4:**
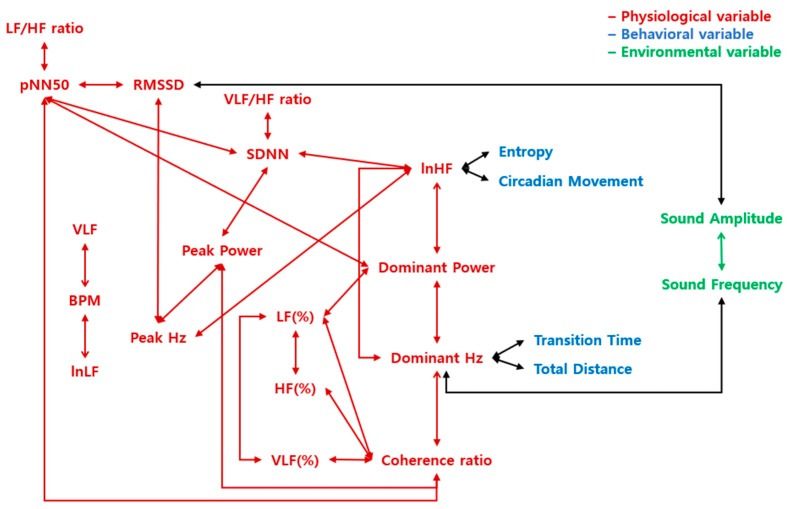
A schematic representation of correlations that demonstrate the differences in arousal of emotions. The letters in red indicate physiological variables, blue indicate behavioral variables, and green indicate environmental variables. The arrows represent the correlation between the two variables. The red arrows represent the correlations within physiological variables, the green arrows represent the correlations within environmental variables, and the black arrows represent the correlations between the different construct variables.

The significant result of statistics that distinguish the three levels of arousal were presented in Table 4, Table 5, Table 6, Table 7, Table 8, Table 9, Table 10, Table 11, Table 12, Table 13, Table 14, Table 15, Table 16, Table 17, Table 18, Table 19, Table 20 and Table 21. The significant result of ANOVA (i.e., *p* < 0.05 in the ANOVA row in the tables) indicates that there is more than one pair of differences among the three levels of emotions. However, this result does not indicate which pair is significant. Therefore, the pairs (arousal-neutral, neutral-relaxation, and arousal-relaxation) that show significant differences should be analyzed. This analysis has been commonly referred to as post-hoc analysis. In this study, an independent t-test was used which is a general method of analyzing the difference between two levels (i.e., *p* < 0.05 in the T-test row in the tables). This paper presents only the results of the significant differences between all pairs (all emotions). The correlations varied depending on the level of arousal were divided into the relationships within physiological or environmental variables, the relationships between the physiological and behavioral variables, and between the physiological and the environmental variables (Figure 4). There were many correlations between physiological variables, especially variables such as pNN50, SDNN, lnHF, Dominant Power, Dominant Hz, Peak Hz and Coherence ratio. The relationships between physiological and behavioral variables, which differed according to the level of arousal, were correlations between lnHF and Entropy, lnHF and Circadian Movement, Dominant Hz and Transition Time, and Dominant Hz and Total Distance. The relationships between physiological and environmental variables, which differed according to the level of arousal, were correlations between RMSSD and Sound Amplitude, and Dominant Hz and Sound Frequency. There was a correlation between sound amplitude and sound frequency. There was no correlation between the behavioral variables.

**Figure 5:**
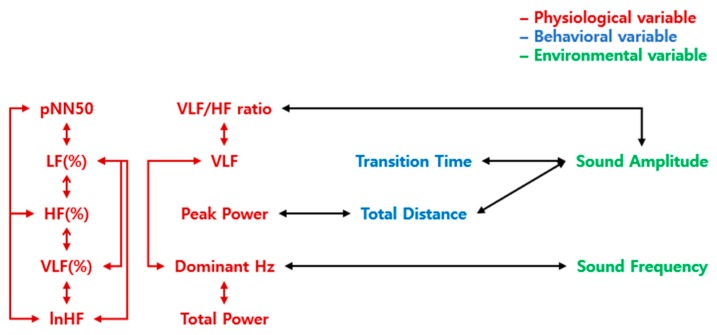
A schematic representation of correlations that distinguish the differences in valence emotions. The letters in red indicate physiological variables, blue indicate behavioral variables, and green indicate environmental variables. The arrows represent the correlation between the two variables. The red arrows represent the correlations within physiological variables, the green arrows represent the correlations within environmental variables, and the black arrows represent the correlations between the different construct variables.

Significant correlations and the statistics which distinguish the three levels of valence were presented in Table 22, Table 23, Table 24, Table 25, Table 26, Table 27, Table 28, Table 29, Table 30, Table 31 and Table 32. Correlations that varied according to the level of valence were classified into the relationships within physiological variables, the relationships between the physiological and behavioral variables, the physiological and environmental variables, and between the behavioral and the environmental variables (Figure 5). There were fewer relationships within the physiological variables in the result of valence than the result of the arousal. The relationship between physiological and behavioral variables was only significant between peak power and total distance. The significant correlations between physiological and environmental variables were VLF/HF ratio and Sound Amplitude, and the relationship between Dominant Hz and Sound Frequency.

## 4. Discussion and Conclusions

The embodied emotion has differed from the previous view of emotion and it has importantly considered interactions among body, behavior, and environment. Therefore, this study was to recognize the embodied emotion by analyzing correlations among physiological changes, behavior, and environment. The physiological responses were determined by cardiovascular responses in this study. The autonomic nervous system (ANS) has been monitored to recognize emotions in many previous studies [44,45,46]. The behavioral patterns of individuals were determined by features, which were analyzed by GPS (global positioning system) locations, according to suppose that lifestyle patterns were associated with emotion [21]. The amplitude and frequency components of ambient sound were considered as the environmental factors, based on environmental factors, particularly ambient sound, which has been related to emotion and physiological arousal in daily life [25,26].

This study verified that there were differences between interactions that determine the arousal and valence in emotion by analyzing an individual’s life-log data. There were more connections between the physiological variables in the result of arousal (Figure 4) than the valence results (Figure 5). In addition, there was no direct connection between behavioral and environmental variables, while both behavioral and environmental variables were associated with physiological variables, as shown in Figure 4. These relationships between physiological and behavioral variables were also more pronounced in the arousal than the valence. These results showed that the autonomic nervous system response has been highly related to physiological arousal [47,48,49,50]. Coherence ratio, an indicator of physiological coherence, has been associated with VLF, LF, an indicator of sympathetic activation, and VLF (%), an indicator of parasympathetic activation. This was consistent with previous theories that physiological coherence has been determined by the way the sympathetic and parasympathetic nerves have been controlled [51]. In addition, lnHF, another indicator of parasympathetic activity, was linked to dominant rhythms (Dominant Power, Dominant Hz) in cardiac activity, which were also connected to the Coherence ratio. This suggests that cardiac activity varies with the degree of activation of parasympathetic nerves and which might be associated with physiological coherence. This association between the autonomic nervous system and physiological coherence was also connected with the indicators of the regularity of life patterns (Entropy, Circadian Movement) and movement patterns (Transition Time, Total Distance). These results suggested that the physiological homeostasis coincides with the behavioral homeostasis according to the polyvagal theory [51,52]. The relationships between physiological variables and ambient noise (Sound Amplitude and Sound Frequency) were consistent with the results that ambient noise has been related to the arousal in the previous studies [25,26].

The relationships among the physiological, behavioral, and environmental variables were more systemic in the result of valence, as shown in Figure 5. It means that there was a connection between the body-behavior-environment in the valence results, compared with arousal results that the behavioral and environmental variables were only connected to physiological variables, respectively. It seems that the conscious and cognitive judgment processes have been necessary to determine the valence level of emotion compared with the arousal level of emotion, which is determined by unconscious and autonomic physiological control [53].

In summary, the arousal levels of embodied emotion were represented by the more prominent interactions with physiological responses, while the valence levels were represented as a balanced relationship among the physiological, behavioral, and environmental variables. These results suggested that the arousal level is an indicator of the regulation of behavioral and physiological homeostasis to cope with the environment, while the valence level indicates the process of cognitive judgment, taking into consideration the environment and behavior. However, because this study was a field test, the experimental controls were less stringent than the laboratory studies. It might be necessary to remove the device, such as when washing hands, in which case, the researchers may not have detected it. Also, cross-validation of these results should be necessary to feasibility and consistency as this study analyzed the data for two weeks for 79 participants for the twenties. Therefore, additional studies should be supported to ensure reproducibility. Nevertheless, this study is valuable because it analyzed practical data. Further, this study serves as an indicator of interpretations, which proves useful in recognizing embodied emotion based on the life-log data in deep-learning or machine-learning.

## Figures and Tables

**Table 1:** An example of the significant causalities analyzed by multiple regression among two weeks data for 79 participants. All assumptions of multiple regression were satisfied. There was no autocorrelation in the residuals (Durbin-Watson value = 2.264). Normality of residuals was satisfied (p-value of Kolmogorov-Smirnov test = 0.728). Homeogeneity of residuals was satisfied (p-value of Breusch-Pagan test = 0.499). Multiple regression was run to predict lnHF from location variance, circadian movement, transition time, total distance, total distance, pNN50, peak hz, and coherence ratio. Only those variables which were not affected by multicollinearity were entered in the multiple-regression (VIF < 10). A significant regression equation was found (*F*(25, 34) = 40.231, *p* < 0.000, Adj.$R^{2}$ = 0.943). Transition time was significant predictor of lnHF. Regression model degrees of freedom: 25, Residual degrees of freedom: 34, Autocorrelation Test - Durbin Watson: 2.264, Kolmogorov-Smirnov Test: *Z* = 0.089, *p =* 0.728, Breusch-Pagan Test: *F* = 0.994, *p* = 0.499.

Dependent Variables	Tests	Statistics			
lnHF	Multiple regression	Determining how well the model fits	Adj. R-square	0.943	
			*F*	40.231	
			Sig.	0.000	
		Statistical significance of the independent variables	Independent variables	Unstandardized coefficients (Beta)	*p*
			(constant)	−10437988370.481	0.661
			Location Variance	0.012	0.687
			Circadian Movement	0.028	0.166
			Transition Time	−0.059	0.076
			Total Distance	0.064	0.116
			pNN50	0.020	0.792
			Peak Hz	0.050	0.115
			Coherence Ratio	0.040	0.247
	Multicollinearity test	Independent variables		VIF	
		Location Variance		2.759	
		Circadian Movement		3.673	
		Transition Time		7.764	
		Total Distance		8.050	
		pNN50		8.528	
		Peak Hz		3.704	
		Coherence Ratio		4.322	

**Table 2:** Descriptive statistics of the standardized coefficients with significant differences between arousal levels.

Variable		Descriptive Statistics of Standardized Coefficients			
Independent	Dependent	Statistic	Arousal	Neutral	Relaxation
BPM	VLF	Mean	−5,745,442,617	−17,308,431,507	8,428,219,435
		SD	270,176,000,000	245,484,000,000	231,354,000,000
pNN50	Dominant Power	Mean	−0.423	−0.613	−0.659
		SD	1.866	2.63	3.945
RMSSD	pNN50	Mean	0.013	0.008	0.01
		SD	0.05	0.041	0.046
SDNN	pNN50	Mean	0.025	0.032	0.034
		SD	0.071	0.099	0.089
SDNN	lnHF	Mean	−0.055	−0.05	−0.141
		SD	0.838	0.931	0.93
SDNN	VLF/HF ratio	Mean	−0.001	−0.003	−0.007
		SD	0.073	0.062	0.082
SDNN	Peak Power	Mean	−0.045	−0.089	−0.056
		SD	0.345	0.53	0.433
LF(%)	VLF(%)	Mean	−0.001	−0.003	−0.006
		SD	0.031	0.053	0.082
LF(%)	HF(%)	Mean	−0.001	−0.003	−0.008
		SD	0.04	0.068	0.098
lnLF	BPM	Mean	0.001	0	−0.001
		SD	0.022	0.017	0.022
lnHF	Entropy	Mean	−1193.169	−140,776.746	14,580.387
		SD	37,241.047	3,206,125.223	642,719.545
lnHF	Circadian Movement	Mean	0	0.001	0
		SD	0.006	0.016	0.004
lnHF	Dominant Hz	Mean	−0.097	−0.112	−0.119
		SD	0.236	0.249	0.254
lnHF	Peak Hz	Mean	0.032	0.04	0.041
		SD	0.08	0.092	0.091
LF/HF ratio	pNN50	Mean	−0.004	−0.006	0
		SD	0.052	0.06	0.067
Dominant Power	lnHF	Mean	−0.041	0.004	0.035
		SD	0.902	0.853	0.872
Dominant Hz	Dominant Power	Mean	−0.008	−0.138	−0.055
		SD	0.791	2.046	1.176
Dominant Hz	Coherence ratio	Mean	−0.042	−0.06	−0.064
		SD	0.259	0.215	0.225
Dominant Hz	Sound Frequency	Mean	−0.002	−0.003	0.003
		SD	0.077	0.051	0.044
Peak Power	Coherence ratio	Mean	0.156	0.127	0.111
		SD	0.456	0.359	0.339
Peak Hz	RMSSD	Mean	−0.075	0.2	−0.013
		SD	1.933	4.134	1.17
Peak Hz	Peak Power	Mean	0.043	0.119	0.216
		SD	1.258	0.878	1.729
Coherence ratio	pNN50	Mean	0.006	−0.002	0.001
		SD	0.077	0.08	0.076
Coherence ratio	VLF(%)	Mean	−105,267,518.5	−2,016,911,138	−48,597,422.56
		SD	15,299,232,245	17,846,539,923	20,110,510,824
Coherence ratio	LF(%)	Mean	−132,068,389.2	−2,021,703,780	−84,532,472.78
		SD	14,109,144,081	17,824,867,958	18,840,262,720
Coherence ratio	HF(%)	Mean	−217,938,238.3	−2,389,073,268	−85,954,745.84
		SD	16,032,965,209	21,954,646,140	22,568,564,779
Coherence ratio	Dominant Hz	Mean	−0.065	−0.074	−0.093
		SD	0.309	0.345	0.325
Transition Time	Dominant Hz	Mean	−0.015	−0.015	0.011
		SD	0.259	0.175	0.194
Total Distance	Dominant Hz	Mean	0.009	0.019	−0.003
		SD	0.182	0.195	0.236
Sound Amplitude	RMSSD	Mean	0.005	0.376	0.02
		SD	0.757	7.924	1.492
Sound Amplitude	Sound Frequency	Mean	0.062	0.078	0.094
		SD	0.245	0.266	0.286
Sound Frequency	Sound Amplitude	Mean	0.075	0.086	0.109
		SD	0.299	0.314	0.337

**Table 3:** Descriptive statistics of the standardized coefficients with significant differences between valence levels.

Variable		Descriptive Statistics of Standardized Coefficients			
Independent	Dependent	Statistic	Positive	Neutral	Negative
Total Distance	Peak Power	Mean	−0.033	−0.128	−0.01
		SD	0.717	2.004	0.7510.751
pNN50	LF(%)	Mean	−118,639,038.5	1,079,730,567	1,891,445,746
		SD	21,820,377,528	18,397,399,419	29,119,919,937
pNN50	HF(%)	Mean	−209,743,703.2	1,227,721,976	2,344,251,772
		SD	25,563,848,513	21,374,411,771	35,526,468,774
VLF(%)	LF(%)	Mean	−0.002	−0.007	−0.001
		SD	0.041	0.081	0.036
VLF(%)	HF(%)	Mean	−0.002	−0.009	−0.002
		SD	0.052	0.104	0.046
LF(%)	VLF(%)	Mean	−0.003	−0.007	−0.001
		SD	0.055	0.088	0.029
LF(%)	HF(%)	Mean	−0.003	−0.009	−0.001
		SD	0.064	0.109	0.041
HF(%)	VLF(%)	Mean	−0.002	−0.006	−0.001
		SD	0.037	0.068	0.021
HF(%)	LF(%)	Mean	−0.002	−0.006	−0.001
		SD	0.036	0.066	0.022
lnHF	VLF(%)	Mean	−6,230,158.347	-808,663,867.5	139,285,674.2
		SD	5,364,187,185	14,013,185,177	5,257,502,233
lnHF	LF(%)	Mean	−5,812,633.013	-910,997,723.4	128,324,064
		SD	5,522,380,316	16,960,721,918	5,205,893,477
lnHF	HF(%)	Mean	−19,292,545.01	−1,142,833,043	160,581,520.7
		SD	6,721,689,128	21,115,017,843	6,435,510,213
VLF/HF ratio	VLF	Mean	−6,771,606,848	4806843674	5,214,155,475
		SD	191,933,000,000	89,138,008,055	97,000,248,564
VLF/HF ratio	Sound Amplitude	Mean	−0.001	0.007	−0.002
		SD	0.051	0.07	0.057
Dominant Hz	VLF	Mean	4,564,168,257	941,743,170	−10,176,921,026
		SD	93,659,975,658	111,036,000,000	224,181,000,000
Dominant Hz	Total Power	Mean	−9,252,548,826	387775431.7	15,059,942,652
		SD	190,801,000,000	222,377,000,000	381,999,000,000
Sound Amplitude	Transition Time	Mean	0.024	−0.007	0.004
		SD	0.327	0.395	0.227
Sound Amplitude	Total Distance	Mean	−0.023	0.023	0.031
		SD	0.49	0.481	0.789
Sound Amplitude	VLF/HF ratio	Mean	−0.011	0.013	0.002
		SD	0.285	0.141	0.304
Sound Frequency	Dominant Hz	Mean	0	−0.01	0.008
		SD	0.143	0.167	0.138

**Table 4:** Results of one-way ANOVA show a significant difference between Arousal-Neutral-Relaxation among variables correlated with BPM analyzed by multiple regression. The difference between the two emotion levels was verified by independent t-test.

Dependent variables	Tests		Statistics	lnLF
BPM	ANOVA		F	3.173
			*p*	0.042
	T-test	Arousal-Neutral	t	1.487
			*p*	0.137
		Neutral-Relaxation	t	0.488
			*p*	0.625
		Arousal-Relaxation	t	−2.358
			*p*	0.018

**Table 5:** Results of one-way ANOVA show a significant difference between Arousal-Neutral-Relaxation among variables correlated with RMSSD analyzed by multiple regression. The difference between the two emotion levels was verified by independent t-test.

Dependent variables	Tests		Statistics	Peak Hz	Sound Amplitude
RMSSD	ANOVA		F	4.067	3.466
			*p*	0.017	0.031
	T-test	Arousal-Neutral	t	-2.346	−2.023
			*p*	0.019	0.043
		Neutral-Relaxation	t	1.964	1.782
			*p*	0.050	0.075
		Arousal-Relaxation	t	1.143	0.401
			*p*	0.253	0.688

**Table 6:** Results of one-way ANOVA show a significant difference between Arousal-Neutral-Relaxation among variables correlated with pNN50 analyzed by multiple regression. The difference between the two emotion levels was verified by independent t-test.

Dependent Variables	Tests		Statistics	SDNN	RMSSD	LF/HF ratio	Coherence Ratio
pNN50	ANOVA		F	5.367	4.233	3.496	3.946
			*p*	0.005	0.015	0.030	0.019
	T-test	Arousal-Neutral	t	−2.200	2.625	0.969	2.502
			*p*	0.028	0.009	0.332	0.012
		Neutral-Relaxation	t	−0.292	−1.109	−2.200	−0.885
			*p*	0.770	0.268	0.028	0.376
		Arousal-Relaxation	t	3.257	−2.004	1.994	−2.092
			*p*	0.001	0.045	0.046	0.036

**Table 7:** Results of one-way ANOVA show a significant difference between Arousal-Neutral-Relaxation among variables correlated with VLF analyzed by multiple regression. The difference between the two emotion levels was verified by independent t-test.

Dependent Variables	Tests		Statistics	BPM
VLF	ANOVA		F	3.109
			*p*	0.045
	T-test	Arousal-Neutral	t	1.032
			*p*	0.302
		Neutral-Relaxation	t	−2.518
			*p*	0.012
		Arousal-Relaxation	t	1.681
			*p*	0.093

**Table 8:** Results of one-way ANOVA show a significant difference between Arousal-Neutral-Relaxation among variables correlated with VLF(%) analyzed by multiple regression. The difference between the two emotion levels was verified by independent t-test.

Dependent Variables	Tests		Statistics	LF(%)	Coherence Ratio
VLF(%)	ANOVA		F	3.597	3.813
			*p*	0.027	0.022
	T-test	Arousal-Neutral	t	1.022	2.793
			*p*	0.307	0.005
		Neutral-Relaxation	t	1.117	−2.338
			*p*	0.264	0.019
		Arousal-Relaxation	t	−2.604	0.096
			*p*	0.009	0.924

**Table 9:** Results of one-way ANOVA show a significant difference between Arousal-Neutral-Relaxation among variables correlated with LF(%) analyzed by multiple regression. The difference between the two emotion levels was verified by independent t-test.

Dependent Variables	Tests		Statistics	Coherence Ratio
LF(%)	ANOVA		F	4.166
			*p*	0.016
	T-test	Arousal-Neutral	t	2.905
			*p*	0.004
		Neutral-Relaxation	t	−2.413
			*p*	0.016
		Arousal-Relaxation	t	0.086
			*p*	0.931

**Table 10:** Results of one-way ANOVA show a significant difference between Arousal-Neutral-Relaxation among variables correlated with HF(%) analyzed by multiple regression. The difference between the two emotion levels was verified by independent t-test.

Dependent Variables	Tests		Statistics	LF(%)	Coherence Ratio
HF(%)	ANOVA		F	3.375	4.056
			*p*	0.034	0.017
	T-test	Arousal-Neutral	t	1.023	2.841
			*p*	0.306	0.005
		Neutral-Relaxation	t	1.050	−2.376
			*p*	0.294	0.018
		Arousal-Relaxation	t	−2.550	0.204
			*p*	0.011	0.838

**Table 11:** Results of one-way ANOVA show a significant difference between Arousal-Neutral-Relaxation among variables correlated with lnHF analyzed by multiple regression. The difference between the two emotion levels was verified by independent t-test.

Dependent Variables	Tests		Statistics	SDNN	Dominant Power
lnHF	ANOVA		F	4.967	3.343
			*p*	0.007	0.035
	T-test	Arousal-Neutral	t	−0.150	−1.178
			*p*	0.881	0.239
		Neutral-Relaxation	t	2.256	−0.832
			*p*	0.024	0.406
		Arousal-Relaxation	t	−2.901	2.556
			*p*	0.004	0.011

**Table 12:** Results of one-way ANOVA show a significant difference between Arousal-Neutral-Relaxation among variables correlated with VLF/HF ratio analyzed by multiple regression. The difference between the two emotion levels was verified by independent t-test.

Dependent Variables	Tests		Statistics	SDNN
VLF/HF ratio	ANOVA		F	3.417
			*p*	0.033
	T-test	Arousal-Neutral	t	0.474
			*p*	0.636
		Neutral-Relaxation	t	1.509
			*p*	0.132
		Arousal-Relaxation	t	−2.475
			*p*	0.013

**Table 13:** Results of one-way ANOVA show a significant difference between Arousal-Neutral-Relaxation among variables correlated with Peak Power analyzed by multiple regression. The difference between the two emotion levels was verified by independent t-test.

Dependent Variables	Tests		Statistics	SDNN	Peak Hz
Peak Power	ANOVA		F	3.013	6.761
			*p*	0.049	0.001
	T-test	Arousal-Neutral	t	2.513	−1.537
			*p*	0.012	0.124
		Neutral-Relaxation	t	−1.600	−1.476
			*p*	0.110	0.140
		Arousal-Relaxation	t	−0.873	3.456
			*p*	0.383	0.001

**Table 14:** Results of one-way ANOVA show a significant difference between Arousal-Neutral-Relaxation among variables correlated with Peak Hz analyzed by multiple regression. The difference between the two emotion levels was verified by independent t-test.

Dependent Variables	Tests		Statistics	lnHF
Peak Hz	ANOVA		F	5.202
			*p*	0.006
	T-test	Arousal-Neutral	t	−2.246
			*p*	0.025
		Neutral-Relaxation	t	−0.179
			*p*	0.858
		Arousal-Relaxation	t	3.058
			*p*	0.002

**Table 15:** Results of one-way ANOVA show a significant difference between Arousal-Neutral-Relaxation among variables correlated with Coherence ratio analyzed by multiple regression. The difference between the two emotion levels was verified by independent t-test.

Dependent Variables	Tests		Statistics	Dominant Hz	Peak Power
Coherence ratio	ANOVA		F	4.194	5.807
			*p*	0.015	0.003
	T-test	Arousal-Neutral	t	1.690	1.553
			*p*	0.091	0.120
		Neutral-Relaxation	t	0.466	1.090
			*p*	0.641	0.276
		Arousal-Relaxation	t	−2.737	−3.311
			*p*	0.006	0.001

**Table 16:** Results of one-way ANOVA show a significant difference between Arousal-Neutral-Relaxation among variables correlated with Dominant Power analyzed by multiple regression. The difference between the two emotion levels was verified by independent t-test.

Dependent Variables	Tests		Statistics	pNN50	Dominant Hz
Dominant Power	ANOVA		F	3.095	3.013
			*p*	0.045	0.049
	T-test	Arousal-Neutral	t	2.117	2.365
			*p*	0.034	0.018
		Neutral-Relaxation	t	0.296	−1.278
			*p*	0.767	0.201
		Arousal-Relaxation	t	−2.342	−1.400
			*p*	0.019	0.162

**Table 17:** Results of one-way ANOVA show a significant difference between Arousal-Neutral-Relaxation among variables correlated with Dominant Hz analyzed by multiple regression. The difference between the two emotion levels was verified by independent t-test.

Dependent Variables	Tests		Statistics	Transition Time	Total Distance	lnHF	Coherence Ratio
Dominant Hz	ANOVA		F	6.946	3.524	3.559	3.252
			*p*	0.001	0.030	0.029	0.039
	T-test	Arousal-Neutral	t	0.014	0.014	1.407	0.626
			*p*	0.989	0.989	0.159	0.531
		Neutral-Relaxation	t	−3.155	−3.155	0.657	1.297
			*p*	0.002	0.002	0.511	0.195
		Arousal-Relaxation	t	3.326	3.326	−2.642	−2.570
			*p*	0.001	0.001	0.008	0.010

**Table 18:** Results of one-way ANOVA show a significant difference between Arousal-Neutral-Relaxation among variables correlated with Entropy analyzed by multiple regression. The difference between the two emotion levels was verified by independent t-test.

Dependent Variables	Tests		Statistics	lnHF
Entropy	ANOVA		F	3.538
			*p*	0.029
	T-test	Arousal-Neutral	t	1.900
			*p*	0.058
		Neutral-Relaxation	t	−1.914
			*p*	0.056
		Arousal-Relaxation	t	1.069
			*p*	0.285

**Table 19:** Results of one-way ANOVA show a significant difference between Arousal-Neutral-Relaxation among variables correlated with Circadian Movement analyzed by multiple regression. The difference between the two emotion levels was verified by independent t-test.

Dependent Variables	Tests		Statistics	lnHF
Circadian Movement	ANOVA		F	3.648
			*p*	0.026
	T-test	Arousal-Neutral	t	−2.202
			*p*	0.028
		Neutral-Relaxation	t	1.621
			*p*	0.105
		Arousal-Relaxation	t	1.614
			*p*	0.107

**Table 20:** Results of one-way ANOVA show a significant difference between Arousal-Neutral-Relaxation among variables correlated with Sound Amplitude analyzed by multiple regression. The difference between the two emotion levels was verified by independent t-test.

Dependent Variables	Tests		Statistics	Sound Frequency
Sound Amplitude	ANOVA		F	5.174
			*p*	0.006
	T-test	Arousal-Neutral	t	−0.878
			*p*	0.380
		Neutral-Relaxation	t	−1.571
			*p*	0.116
		Arousal-Relaxation	t	3.191
			*p*	0.001

**Table 21:** Results of one-way ANOVA show a significant difference between Arousal-Neutral-Relaxation among variables correlated with Sound Frequency analyzed by multiple regression. The difference between the two emotion levels was verified by independent t-test.

Dependent variables	Tests		Statistics	Dominant Hz	Sound Amplitude
Sound Frequency	ANOVA		F	3.314	6.380
			*p*	0.036	0.002
	T-test	Arousal-Neutral	t	0.359	−1.420
			*p*	0.720	0.156
		Neutral-Relaxation	t	−2.788	−1.355
			*p*	0.005	0.175
		Arousal-Relaxation	t	2.141	3.574
			*p*	0.032	0.000

**Table 22:** Results of ANOVA show a significant difference between Positive-Neutral-Negative among variables correlated with VLF analyzed by multiple regression. The difference between the two emotion levels was verified by independent t-test.

Dependent Variables	Tests		Statistics	VLF/HF Ratio	Dominant Hz
VLF	ANOVA		F	3.238	4.067
			*p*	0.039	0.017
	T-test	Positive-Neutral	t	−1.811	0.959
			*p*	0.07	0.338
		Neutral-Negative	t	−0.099	1.409
			*p*	0.921	0.159
		Positive-Negative	t	1.953	−2.698
			*p*	0.051	0.007

**Table 23:** Results of one-way ANOVA show a significant difference between Positive-Neutral-Negative among variables correlated with VLF(%) analyzed by multiple regression. The difference between the two emotion levels was verified by independent t-test.

Dependent Variables	Tests		Statistics	LF(%)	HF(%)	lnHF
VLF(%)	ANOVA		F	3.359	4.107	4.281
			*p*	0.035	0.017	0.014
	T-test	Positive-Neutral	t	1.82	2.166	2.37
			*p*	0.069	0.03	0.018
		Neutral-Negative	t	−2.329	−2.366	−2.083
			*p*	0.02	0.018	0.037
		Positive-Negative	t	1.094	0.913	0.744
			*p*	0.274	0.361	0.457

**Table 24:** Results of one-way ANOVA show a significant difference between Positive-Neutral-Negative among variables correlated with LF(%) analyzed by multiple regression. The difference between the two emotion levels was verified by independent t-test.

Dependent Variables	Tests		Statistics	pNN50	VLF(%)	HF(%)	lnHF
LF(%)	ANOVA		F	3.001	3.872	3.952	4.002
			*p*	0.05	0.021	0.019	0.018
	T-test	Positive-Neutral	t	−1.509	2.394	2.145	2.291
			*p*	0.131	0.017	0.032	0.022
		Neutral-Negative	t	−0.75	−1.997	−2.315	−1.931
			*p*	0.453	0.046	0.021	0.054
		Positive-Negative	t	2.243	0.185	0.861	0.674
			*p*	0.025	0.853	0.389	0.5

**Table 25:** Results of one-way ANOVA show a significant difference between Positive-Neutral-Negative among variables correlated with HF(%) analyzed by multiple regression. The difference between the two emotion levels was verified by independent t-test.

Dependent Variables	Tests		Statistics	pNN50	VLF(%)	LF(%)	lnHF
HF(%)	ANOVA		F	3.367	3.938	3.438	4.057
			*p*	0.035	0.02	0.032	0.017
	T-test	Positive-Neutral	t	−1.548	2.415	1.941	2.294
			*p*	0.122	0.016	0.052	0.022
		Neutral-Negative	t	−0.857	−2.014	−2.244	−1.946
			*p*	0.392	0.044	0.025	0.052
		Positive-Negative	t	2.387	0.204	0.953	0.739
			*p*	0.017	0.839	0.341	0.46

**Table 26:** Results of one-way ANOVA show a significant difference between Positive-Neutral-Negative among variables correlated with VLF/HF ratio analyzed by multiple regression. The difference between the two emotion levels was verified by independent t-test.

Dependent Variables	Tests		Statistics	Sound Amplitude
VLF/HF ratio	ANOVA		F	3.149
			*p*	0.043
	T-test	Positive-Neutral	t	−2.552
			*p*	0.011
		Neutral-Negative	t	1.008
			*p*	0.313
		Positive-Negative	t	1.28
			*p*	0.201

**Table 27:** Results of one-way ANOVA show a significant difference between Positive-Neutral-Negative among variables correlated with Peak Power analyzed by multiple regression. The difference between the two emotion levels was verified by independent t-test.

Dependent Variables	Tests		Statistics	Total Distance
Peak Power	ANOVA		F	3.186
			*p*	0.041
	T-test	Positive-Neutral	t	1.991
			*p*	0.047
		Neutral-Negative	t	−1.81
			*p*	0.07
		Positive-Negative	t	0.858
			*p*	0.391

**Table 28:** Results of one-way ANOVA show a significant difference between Positive-Neutral-Negative among variables correlated with Dominant Hz analyzed by multiple regression. The difference between the two emotion levels was verified by independent t-test.

Dependent Variables	Tests		Statistics	Sound Frequency
Dominant Hz	ANOVA		F	3.826
			*p*	0.022
	T-test	Positive-Neutral	t	1.784
			*p*	0.075
		Neutral-Negative	t	−2.671
			*p*	0.008
		Positive-Negative	t	1.473
			*p*	0.141

**Table 29:** Results of one-way ANOVA show a significant difference between Positive-Neutral-Negative among variables correlated with Total Power analyzed by multiple regression. The difference between the two emotion levels was verified by independent t-test.

Dependent Variables	Tests		Statistics	Dominant Hz
Total Power	ANOVA		F	3.305
			*p*	0.037
	T-test	Positive-Neutral	t	−1.26
			*p*	0.208
		Neutral-Negative	t	−1.055
			*p*	0.291
		Positive-Negative	t	2.473
			*p*	0.013

**Table 30:** Results of one-way ANOVA show a significant difference between Positive-Neutral-Negative among variables correlated with Total Distance analyzed by multiple regression. The difference between the two emotion levels was verified by independent t-test.

Dependent Variables	Tests		Statistics	Sound Amplitude
Total Distance	ANOVA		F	4.284
			*p*	0.014
	T-test	Positive-Neutral	t	−2.509
			*p*	0.012
		Neutral-Negative	t	−0.28
			*p*	0.779
		Positive-Negative	t	2.473
			*p*	0.013

**Table 31:** Results of one-way ANOVA show a significant difference between Positive-Neutral-Negative among variables correlated with Transition Time analyzed by multiple regression. The difference between the two emotion levels was verified by independent t-test.

Dependent Variables	Tests		Statistics	Sound Amplitude
Transition Time	ANOVA		F	3.66
			*p*	0.026
	T-test	Positive-Neutral	t	2.333
			*p*	0.02
		Neutral-Negative	t	−0.769
			*p*	0.442
		Positive-Negative	t	−1.852
			*p*	0.064

**Table 32:** Results of one-way ANOVA show a significant difference between Positive-Neutral-Negative among variables correlated with Sound Amplitude analyzed by multiple regression. The difference between the two emotion levels was verified by independent t-test.

Dependent Variables	Tests		Statistics	VLF/HF Ratio
Sound Amplitude	ANOVA		F	6.852
			*p*	0.001
	T-test	Positive-Neutral	t	−3.389
			*p*	0.001
		Neutral-Negative	t	2.918
			*p*	0.004
		Positive-Negative	t	−0.374
			*p*	0.708
